# *De Novo* Transcriptome Sequence Assembly and Identification of AP2/ERF Transcription Factor Related to Abiotic Stress in Parsley (*Petroselinum crispum*)

**DOI:** 10.1371/journal.pone.0108977

**Published:** 2014-09-30

**Authors:** Meng-Yao Li, Hua-Wei Tan, Feng Wang, Qian Jiang, Zhi-Sheng Xu, Chang Tian, Ai-Sheng Xiong

**Affiliations:** State Key Laboratory of Crop Genetics and Germplasm Enhancement, College of Horticulture, Nanjing Agricultural University, Nanjing, China; University of Western Sydney, Australia

## Abstract

Parsley is an important biennial Apiaceae species that is widely cultivated as herb, spice, and vegetable. Previous studies on parsley principally focused on its physiological and biochemical properties, including phenolic compound and volatile oil contents. However, little is known about the molecular and genetic properties of parsley. In this study, 23,686,707 high-quality reads were obtained and assembled into 81,852 transcripts and 50,161 unigenes for the first time. Functional annotation showed that 30,516 unigenes had sequence similarity to known genes. In addition, 3,244 putative simple sequence repeats were detected in curly parsley. Finally, 1,569 of the identified unigenes belonged to 58 transcription factor families. Various abiotic stresses have a strong detrimental effect on the yield and quality of parsley. AP2/ERF transcription factors have important functions in plant development, hormonal regulation, and abiotic response. A total of 88 putative AP2/ERF factors were identified from the transcriptome sequence of parsley. Seven AP2/ERF transcription factors were selected in this study to analyze the expression profiles of parsley under different abiotic stresses. Our data provide a potentially valuable resource that can be used for intensive parsley research.

## Introduction

Parsley (*Petroselinum crispum* L.) is a biennial Apiaceae species that is native to the Mediterranean coast and widely cultivated in Europe and Japan. Parsley is subdivided into three principal types according to cultivation: curly leaf type (subspecies *crispum*), plain leaf type (subspecies *neapolitanum*), and “Hamburg” root parsley (subspecies *tuberosum*). The curly leaf and plain leaf types are cultivated for their foliage, whereas root parsley is grown as a root vegetable [1]. Parsley is widely utilized in the cosmetic, medicinal, and food industries because it is an excellent source of phenolic compounds, volatile oils, vitamins, and nutrients [2]–[4].

Global challenges, such as climate change, environmental degradation, and toxic waste, subject plants to various stresses during growth. Drought, high salinity, and extreme temperature are the major limiting factors of higher plant growth and production. Numerous genes in higher plants are activated in response to these abiotic stresses [5]. Genes can either directly respond to stresses or regulate the expression of other genes and signal transcription [6], [7]. Transcription factors function in gene expression by combining DNA-binding and *cis*-acting elements [8], [9]. Many transcription factors, such as AP2/ERF, NAC, bZIP, and WRKY, are related to stress resistance in plants [10]–[13]. These transcription factors interact to regulate gene expression and form complex gene regulatory networks [9], [14], [15]. Up to now, little is known about the abiotic stress tolerance of parsley. Cormack [16] isolated two WRKY transcription factors from parsley using the yeast one-hybrid system. Weisshaar [17] cloned three bZIP genes from parsley and found that these genes are involved in the response to environmental changes and disease invasion. However, almost no AP2/ERF members have been identified in parsley. AP2/ERF was one of the largest transcription factor families in higher plants and has received much attention in recent years. This family can be further classified into four subfamiles: ERF, DREB, AP2, RAV [18]–[20]. Numerous reports have demonstrated that the family members can regulate plant responses to abiotic stresses [21], [22]. *JERF3*, an ERF member in tomato, can be induced by abscisic acid, ethylene, jasmonic acid, and low temperature; ectopic overexpression of *JERF3* in transgenic tobacco enhances salt tolerance [23]. A DREB-type gene *LsDREB2A*, was isolated from lettuce, can increased the tolerance of salt stress in transgenic plants [24].

As of this writing, research on parsley has principally focused on essential oil content [25]–[27] and flavonoid products [28], but information on molecular biology and gene function is lacking. No genome-sequenced species in the Apiaceae family has limited the research. As far as we know, only three transcriptome sequence information has obtained from celery [29], [30] and carrot [31] in the Apiaceae family, which were belonged to Apium and Daucus genus, respectively. The limited resources cannot provide more help to study the parsley, which is belonged to Petroselinum genus. RNA-Seq is a feasible and economical modern sequencing technology for obtaining transcriptomic data in a short time. This technology can detect new transcripts that correspond to existing genomic sequences; it can also be used to generate sequence resources for gene discovery, expression, and annotation, and for discovering simple sequence repeats (SSRs) and single nucleotide polymorphisms (SNPs) in non-model organisms without a reference genome [32]–[34]. RNA-Seq has been used to obtain transcriptomic data for an increasing number of organisms, such as tobacco [35], grapevine [36], sunflower [37], and sweet potato [38]. This method is convenient for intensive studies in molecular biology. In the present study, we performed the first comprehensive analysis of the transcriptome of parsley using Illumina paired-end sequencing technology, which can provide valuable resources for intensive parsley research. The AP2/ERF gene family was also analyzed based on the obtained data. Some genes in the AP2/ERF family were isolated, and their relation to abiotic stress response was detected. The results of this study could be used to analyze the molecular mechanism underlying the stress tolerance of parsley.

## Methods and Materials

### Plant materials

The curly parsley cultivar (*P. crispum* L. subsp. *crispum*) was used as plant material (Figure S1). Seeds were sown in a pot containing a soil/vermiculite mixture (3∶1) in a controlled-environment growth chamber under a 16 h/8 h photoperiod at 25°C/16°C day/night cycle. After 10 weeks, leaves, stems, and roots were collected, immediately frozen in liquid nitrogen, and then stored at –70°C for RNA extraction.

### RNA isolation and library preparation for sequencing

Total RNA of mixed sample was extracted using the RNAsimple total RNA kit according to the manufacturer’s instructions (Tiangen, Beijing, China). The quantity and quality of the extracted RNA were verified by gel electrophoresis and spectrophotometry (Nanodrop-ND-1000 spectrophotometer, Nanodrop Technologies Inc., Delaware, USA). mRNA was concentrated using oligo(dT) magnetic adsorption and then broken into fragments, which were used as templates to synthesize first- and second-strand cDNA. The double-stranded cDNA was further purified using the QiaQuick PCR extraction kit (Qiagen, Hilden, Germany), resolved for final reparation and poly(A) addition, and then connected with different sequencing adapters. A library with a suitable insert length (300 bp to 500 bp) was sequenced by Biomarker technologies Co., Ltd. (Beijing, China) using the Illumina HiSeqTM 2000. The sequence data of parsley were submitted to NCBI Sequence Read Archive (http://trace.ncbi.nlm.nih.gov/Traces/sra/) under the accession number SRA111430.

### *De novo* assembly

The raw reads were first cleaned by filtering adaptor sequences and low-quantity reads (more than 50% of bases with Q-value ≤20). For *de* *novo* assembly, the clean reads were mapped back to the contigs by Trinity [39] with the parameters set at a similarity of 90%. Subsequently, the contigs were assembled to construct transcripts with pair-end information and clustered to obtain unigenes. Open reading frames (ORFs) were identified using the Getorf program [40].

### Putative SSR screening

All detected unigenes were used for screening putative SSRs by MIcroSAtellite tool (http://pjrc.ipk-gatersleben.de/misa/) [41]. The putative SSRs contained motifs with one to six nucleotides, and the parameters of contiguous repeat units were set for mono-, di-, tri-, tetra-, penta-, and hexa-nucleotide motifs with a minimum of 10, 6, 5, 5, 5, and 5 repeats, respectively.

### Functional annotation

A sequence similarity search was performed against seven databases to investigate the putative functions of the unigenes based on sequence or domain alignment. All unigenes were compared with genes in the NCBI non-redundant protein (Nr), NCBI Non-redundant Nucleotide (Nt), Swiss-Prot, TrEMBL, Gene Ontology (GO, http://www.geneontology.org/), Clusters of Orthologous Groups (COG), and Kyoto Encyclopedia of Genes and Genomes (KEGG) databases [42]–[44]. Homology search against the Nr database was performed to identify top-hit species by BLASTx with a cut-off E-value of 1e-5. Blast2GO [45] was employed to obtain the functional classification, and WEGO [46] was used to perform the distribution of GO classification.

### Transcription abundance analysis

The transcription abundance of each unigene in the curly parsley library was measured by calculating read density as reads per kilobase of the transcript per million mapped reads (RPKM) to the transcriptome [47]. The RPKM indicates the expression level of each unigene by normalizing the counts of sequenced reads mapped to a gene against the transcript length and the sequencing depth.

### Multiple sequence alignments and phylogenetic analyses of AP2/ERF transcription factors

HMMER and local BLAST were used to screen the transcription factors with E-values below 1e-5. Sequence alignments of the AP2/ERF proteins in parsley and *Arabidopsis* were performed with ClustalW [48] using default parameters. A phylogenetic tree was constructed with MEGA 5.0 [49] using the neighbor-joining method with the bootstrap was set to 1,000.

### Abiotic stress treatments and quantitative reverse transcription-polymerase chain reaction (qRT–PCR)

Half of the two-month-old curly parsley seedlings were transferred to growth chambers set at 4°C or 38°C, which represented low and high temperature stress treatments. The other seedlings were irrigated with double-distilled H_2_O (control), 200 mM NaCl (salt treatment), and 20% polyethylene glycol 6000 (drought treatment). Young leaf samples were collected at 0, 1, 2, 4, 8, and 24 h after the different stress treatments. Total RNA was isolated using the total RNA kit (RNAsimply, Tiangen, Beijing, China) and then reverse transcribed into cDNA using the PrimeScript RT reagent Kit (TaKaRa, Dalian, China). qRT–PCR was performed using MyiQ Single color Real-Time PCR Detection System (Bio-rad, Hercules, CA, USA) with SYBR Premix Ex-Taq (TaKaRa, Dalian, China). The PCR conditions were as follows: 95°C for 30 s; 40 cycles of 95°C for 5 s and 60°C for 30 s; and 65°C for 15 s. The primers of unigenes and *actin* are listed in Table S1. The experiments were repeated three bio-replicates and tech-replicates, and *actin* was used as a reference gene. The expression levels of the unigenes were calculated by the 2^−ΔΔCT^ method [50].

## Results

### Sequencing, *de* *novo* assembly, and sequence analysis of parsley

**Figure 1:**
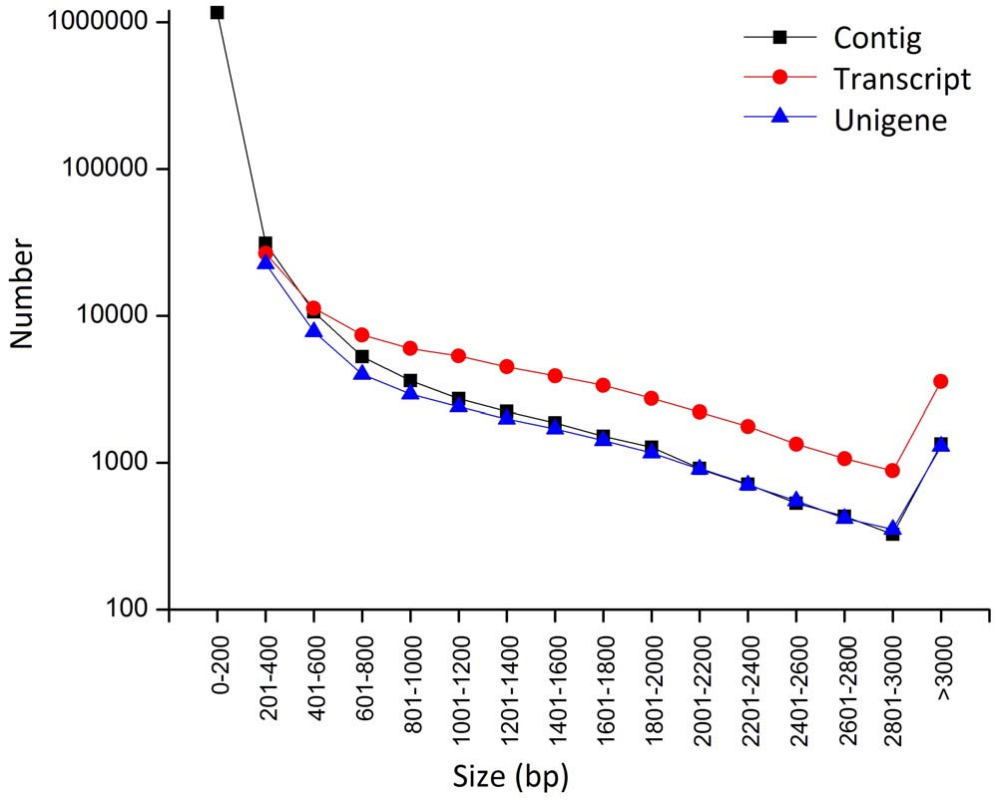
Size distribution of the assembled contigs, transcripts, and unigenes.

A cDNA library of curly parsley was constructed for transcriptome sequencing. Sequence data of 4.78 Gb were generated, and 23,686,707 reads were obtained with 95.56% Q20 bases as high-quality reads. The high-quality reads were assembled into 1,224,381 contigs with an N50 length of 126 bp and a mean length of 87 bp by Trinity [39]. The contigs were further assembled into 81,852 transcripts and clustered into unigenes using a paired-end sequencing strategy. A total of 50,161 unigenes were obtained. These unigenes had lengths in the range of 201 bp to 15,178 bp, an N50 length of 1,344 bp, and a mean length of 802 bp. Most of the unigenes (54.41% in curly parsley) had lengths in the range of 200 bp to 500 bp. Up to 9,982 (19.90%) unigenes had lengths in the range of 500 bp to 1,000 bp, and 12,888 (25.59%) unigenes had lengths of >1,000 bp. The size distributions of the contigs, transcripts, and unigenes are shown in Figure 1. Getorf [40] was used to find and extract the ORFs of all the unigenes to obtain the coding and protein sequences. Up to 49,946 putative coding sequences were identified. These sequences can be used for gene cloning and functional verification.

### Selection and analysis of putative SSRs

SSRs are repeating DNA sequences of 1 bp to 6 bp in both coding and non-coding regions of the genome [51]. SSRs are commonly used in gene mapping because of their high polymorphism, wide distribution in the genome, and easy operation. In this study, we identified 3,244 putative SSRs in 2,643 curly parsley unigenes, among which 473 had more than one SSR. Up to 299 unigenes occurred in compound formation.

**Figure 2:**
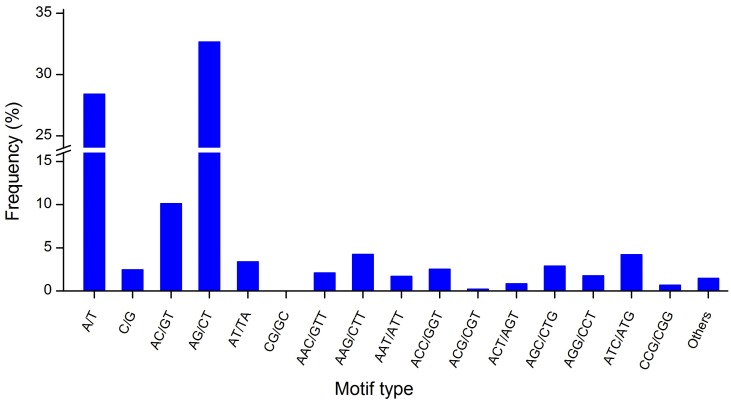
Frequency distribution of SSRs according to motif type.

All putative SSRs had different lengths between different repeat types (Table 1). The di-nucleotide SSRs comprised the largest fraction (46.24%), followed by mono-nucleotide (30.89%) and tri-nucleotide (21.39%) SSRs. Other types of SSRs (tetra-, penta-, and hexa-nucleotide repeats) had a frequency of less than 1.5%. The frequencies of SSR motif types were also analyzed (Figure 2). Most of the mono-nucleotides were of the A/T type, accounting for 28.42% of all SSRs and were almost 15-fold higher than the C/G type. Di-nucleotide repeat motifs were divided into four classes; the most abundant types were AG/CT and AC/GT, which accounted for 32.68% and 10.14% of all SSRs, respectively. Tri-nucleotide repeat motifs were divided into 10 categories; the most abundant types were AAG/CTT and ATC/ATG. The formations of mono-, di-, and tri-nucleotide repeat types comprised numerous A and T repeat elements, showing a strong base preference.

**Table 1:** SSR types in curly parsley transcriptome.

Repeat type	Repeat numbers												Total	%
	5	6	7	8	9	10	11	12	13	14	15	>15		
Mono-nucleotide	0	0	0	0	0	393	186	130	67	43	48	135	1002	30.89
Di-nucleotide	0	460	297	213	224	224	79	2	0	0	0	1	1500	46.24
Tri-nucleotide	408	168	102	13	1	0	0	0	1	0	0	1	694	21.39
Tetra-nucleotide	26	11	2	1	0	0	0	0	0	0	0	0	40	1.23
Penta-nucleotide	4	0	0	0	0	0	0	0	0	0	0	0	4	0.12
Hexa-nucleotide	0	1	1	1	0	1	0	0	0	0	0	0	4	0.12
Total	438	640	402	228	225	618	265	132	68	43	48	137	3244	100
%	13.5	19.73	12.39	7.03	6.94	19.05	8.17	4.07	2.1	1.33	1.48	4.22	100	

### Functional classification of curly parsley unigenes

**Figure 3:**
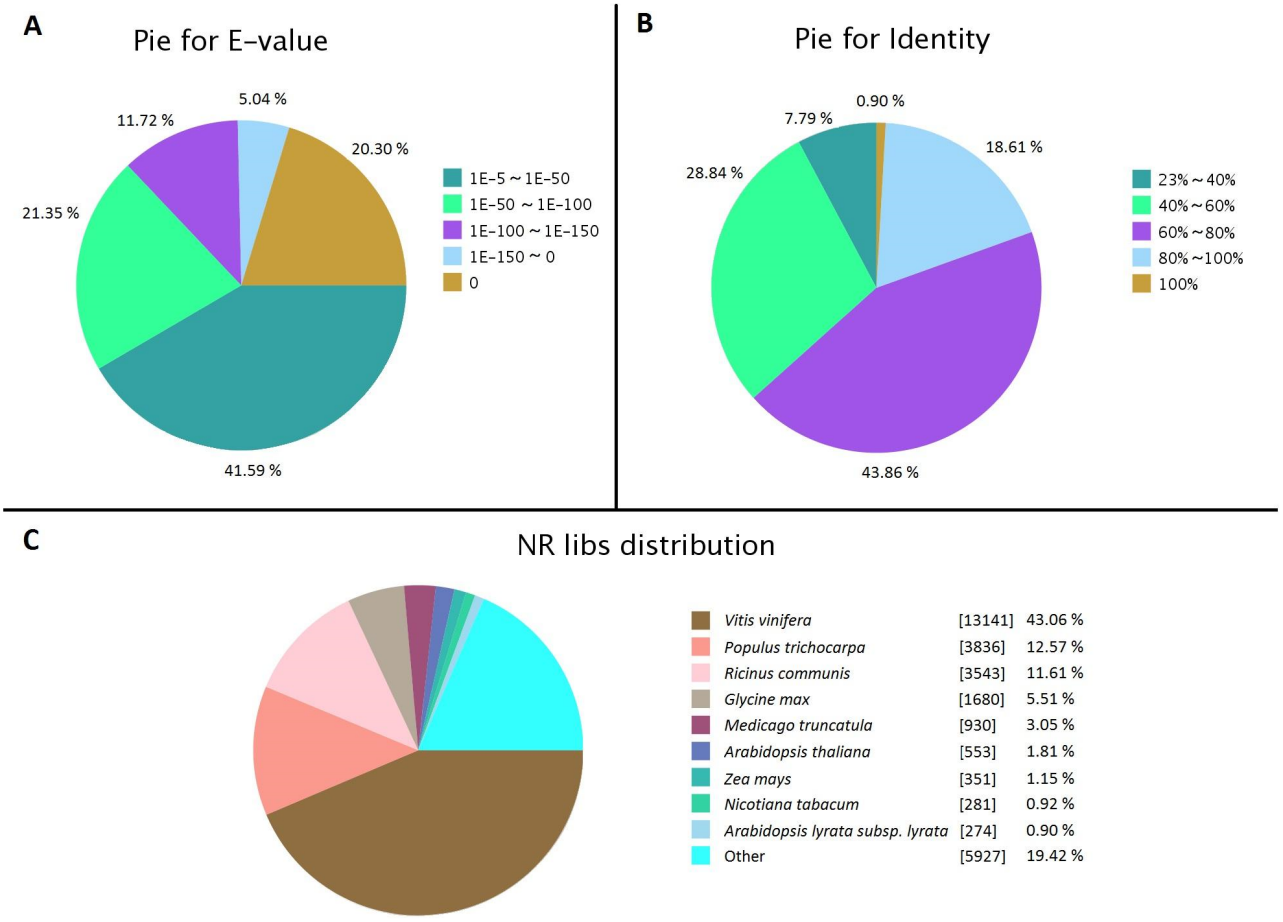
Characteristics of homology of curly parsley unigenes. (A) E-value distribution of BLASTx hits against Nr database for unigenes; (B) Sequence similarity distribution of the best Blastx hits for all unigenes; (C) Proportion of unigenes matched to each species by BLASTx; the top 9 species are indicated.

A sequence similarity search was performed based on sequence- and domain-based alignments to functionally annotate the parsley transcriptome. All unigenes were searched against seven public databases. The sequences that appeared on each database are listed in Table 2. All unigenes were first compared with genes in the NCBI non-redundant database based on sequence alignment using BLASTx with a cut-off E-value of 1e-5. Up to 30,516 unigenes (60.84% of all assembled unigenes) had sequence similarity to known genes. The distributions of E-value and sequence similarity were comparable, with 58.41% (E-value between 0 and 1e-50) and 19.51% (sequence similarity between 80% and 100%) showing very strong homology, respectively (Figures 3A and 3B). For species distribution of the best match, *P. crispum* showed the highest similarity to *Vitis vinifera* (43.06%), followed by *Populus trichocarpa* (12.57%) and *Ricinus comunis* (11.61%) (Figure 3C). The more detailed information of annotations was represented in Table S2.

**Table 2:** Functional annotation of curly parsley unigenes by sequence similarity search.

Annotated Databases	Annotated Number	300≦length<1000	length≧1000
Nr	30516	13644	12460
Nt	22871	8948	11225
TrEMBL	30410	13612	12465
Swissprot	23432	9661	10773
GO	26149	11167	11493
COG	9469	3188	5438
KEGG	6569	2519	3139
Total Annotated	31658	14256	12513

**Figure 4:**
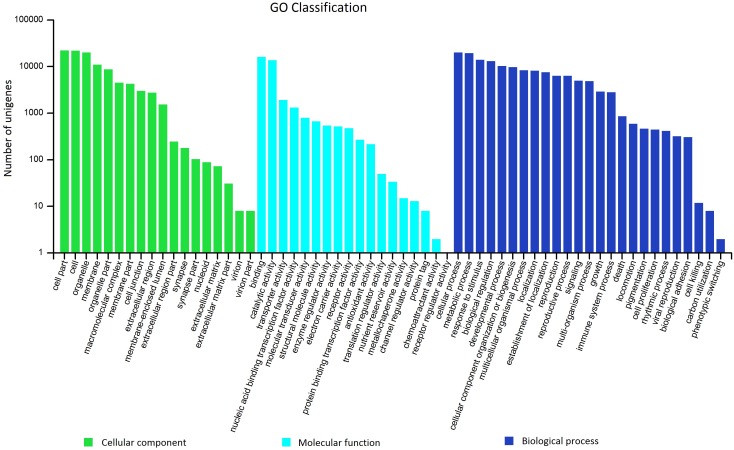
GO classification of assembled unigenes of *P. crispum*. The classifications are shown in 3 principal categories and 61 functional groups.

**Figure 5:**
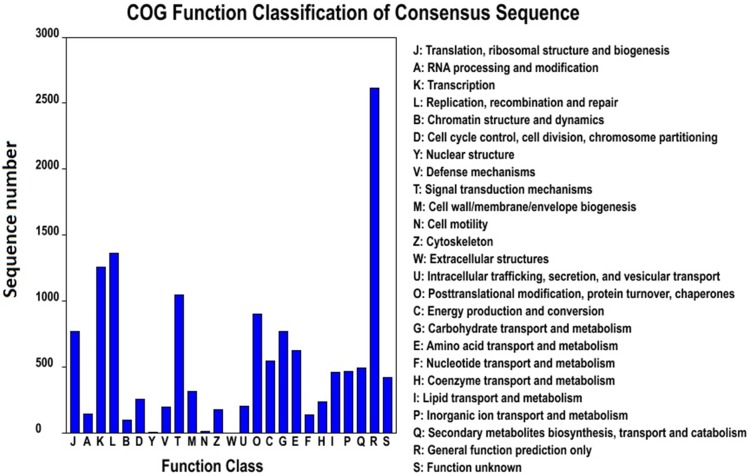
COG function classification of assembled unigenes of *P. crispum*.

**Figure 6:**
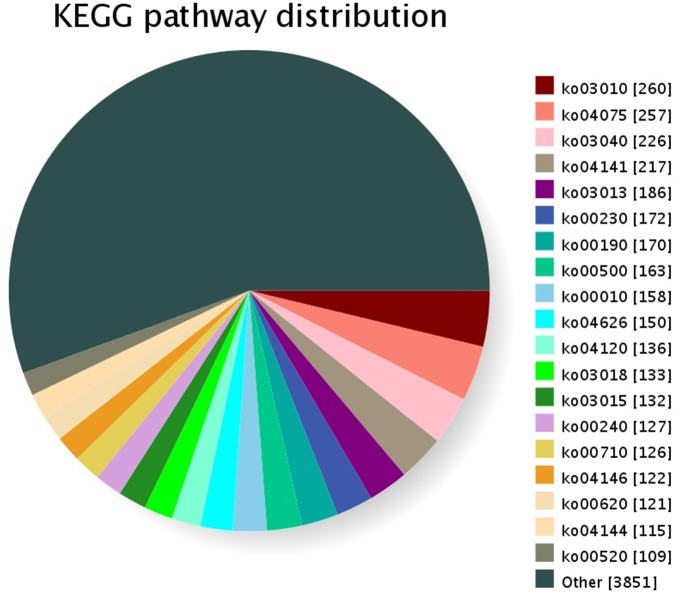
Distribution of each KEGG pathway number against the KEGG database. Each color represents a KEGG pathway. The top 19 KEGG pathways are indicated. The number of unigenes mapped in each pathway is indicated with brackets. The abbreviations represent the pathways as follows: ko03010: Ribosome; ko04075: Plant hormone signal transduction; ko03040: Spliceosome; ko04141: Protein processing in endoplasmic reticulum; ko03013: RNA transport; ko00230: Purine metabolism; ko00190: Oxidative phosphorylation; ko00500: Starch and sucrose metabolism; ko00010: Glycolysis/Gluconeogenesis; ko04626: Plant-pathogen interaction; ko04120: Ubiquitin mediated proteolysis; ko03018: RNA degradation; ko03015: mRNA surveillance pathway; ko00240: Pyrimidine metabolism; ko00710: Carbon fixation in photosynthetic organisms; ko04146: Peroxisome; ko00620: Pyruvate metabolism; ko04144: Endocytosis; ko00520: Amino sugar and nucleotide sugar metabolism.

GO was used to classify the unigenes into functional categories by Blast2GO. A total of 26,149 unigenes were annotated and classified into 3 gene ontology categories and 61 functional groups (Figure 4). In the “cellular compound” category, “cell part” (22.03%) was the most dominant group, followed by “cell” (21.82%) and “organelle” (19.96%). Under the “molecular function” category, “binding” (43.80%) and “catalytic activity” (37.51%) were the most dominant groups. In the “biological process” category, “cellular process” (14.05%), “metabolic process” (13.68%), and “response to stimulus” (9.83%) were the most dominant groups. According to the COG database, 9,469 unigenes were clustered into 25 functional categories (Figure 5). “General function prediction only” (19.43%) was the largest COG category, followed by “replication, recombination, and repair” (10.09%) and “transcription” (9.32%). In addition, all unigenes were searched against the KEGG pathway database. A total of 6,569 unigenes were mapped to 137 pathways. The top 19 KEGG pathways, which contained over 100 unigenes, are shown in Figure 6. “Ribosome” (PATH:ko03010), “plant hormone signal transduction” (PATH:ko04075), and “spliceosome” (PATH:ko03040) were the most dominant pathways.

### Identification of transcription factors in parsley

**Figure 7:**
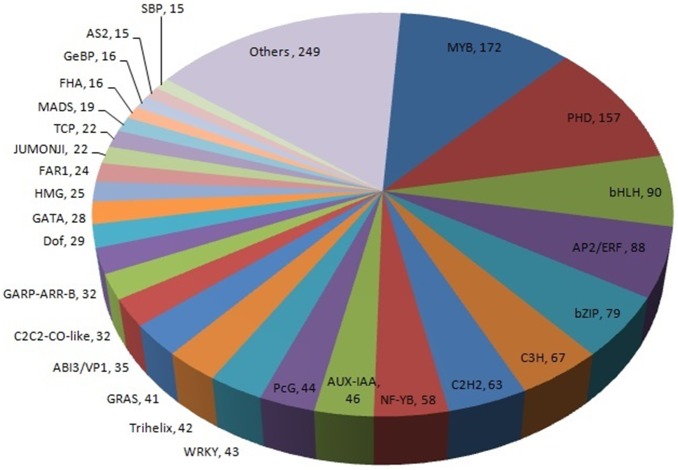
Family distribution of the transcription factors occurring in the curly parsley transcriptome. The number of each transcription factor family members was represented. Families comprising less than 15 transcription factors are classified under others.

HMMER and local BLAST with E-values below 1e-5 were used to screen the transcription factors from curly parsley transcriptome. Transcription factor families were classified according to the Plant Transcription Factor Database (Version 3.0) [52]. Up to 1,569 of the identified unigenes belonged to 58 transcription factor families (Figure 7 and Table S3). The most highly represented transcription factor families were MYB (172 unigenes), PHD (157 unigenes), bHLH (90 unigenes), and AP2/ERF (88 unigenes). Among these members, MYB and bHLH transcription factors may be involved in flavonoid biosynthesis, whereas PHD and AP2/ERF transcription factors may be involved in stress response [53]–[55].

### Phylogenetic relationship of AP2/ERF transcription factors

**Figure 8:**
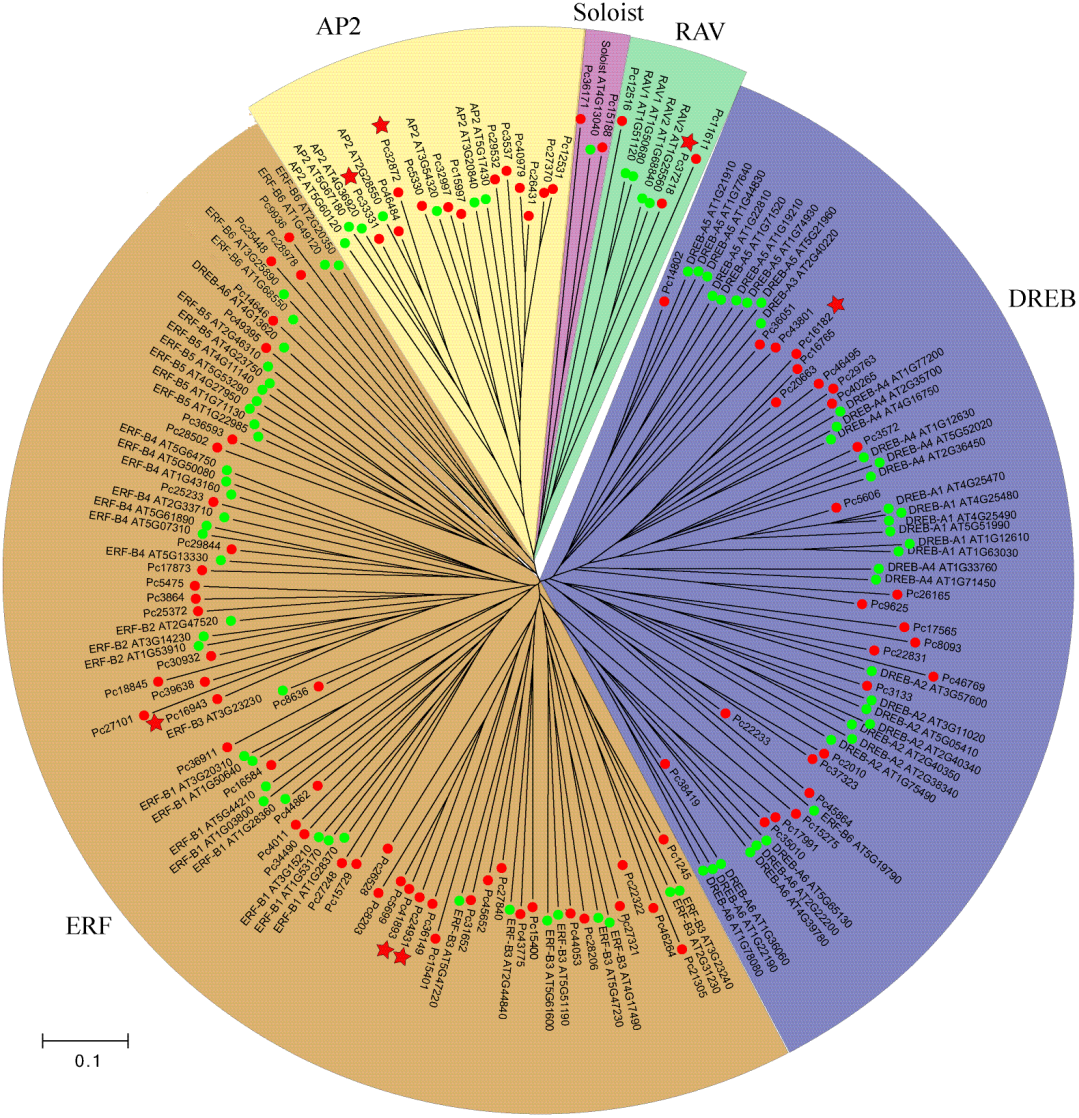
Phylogenetic tree of all AP2/ERF transcription factors from parsley and *Arabidopsis*. All the circles represent the AP2/ERF genes in parsley (red) and *Arabidopsis* (green). The red pentagrams represent the AP2/ERF genes which were selected to detected response to four abiotic stresses.

Transcription factors of the AP2/ERF gene family can be divided into four subgroups (DREB, ERF, RAV, AP2), and Soloist based on the sequence similarity [18]–[20]. To confirm the subfamily classification and analyze the evolutionary relationship between carrot and *Arabidopsis*, we used the AP2/ERF amino acid sequences to generate a phylogenetic tree. As shown in Figure 8, all the 88 AP2/ERFs were classified into five subfamilies with the 49 members in ERF subfamily, 22 members in DREB subfamily, 12 members in AP2 subfamily, 3 members in RAV and 2 members in Soloist. Compared with other species [18], [56]–[60], the AP2/ERF family in parsley seems to have relatively less members (Table 3). The numbers of each subfamily members were varied among different species. ERF is consistently the largest subfamily in these seven plants, followed by DREB, AP2, RAV, and Soloist.

**Table 3:** Summary of the AP2/ERF family factors in parsley, *Arabidopsis*, castor bean, apple, maize, rice and wheat.

Plants	Dicotyledon				Monocotyledon		
Classification	*Petroselinum* *crispum*	*Arabidopsis* *thaliana*	*Ricinus* *communis*	*Malus *×*domestica*	*Zea* *mays*	*Oryzae* *sativa*	*Triticum* *aestivum*
ERFsubfamily	49	65	56	127	107	79	47
DREBsubfamily	22	57	34	68	51	52	57
AP2subfamily	12	18	19	51	22	26	9
RAVsubfamily	3	6	4	6	3	7	3
Soloist	2	1	1	7	1	0	1
Total	88	147	114	259	184	164	117

### Expression analysis of AP2/ERF genes under abiotic stresses

The transcript expression levels of all unigenes in the curly parsley library were estimated by calculating read density as RPKM [47]. The RPKMs of >50% of the unigenes ranged from 1 to 50, and those of >5% of the unigenes were >50 (Table S4).

Many studies have reported that the members of AP2/ERF family genes involved in abiotic stress response [21], [61]. In the present study, seven AP2/ERF transcription factors belonging to four subgroups were selected to detected response to four abiotic stresses (low temperature, high temperature, high salinity, and drought). The genes of *Pc16182*, *Pc16943*, *Pc24931*, and *Pc41893* were belonged to ERF subfamily. The genes of *Pc32872* and *Pc33331* were chose from AP2 subfamily. The gene of *Pc37218* was selected from RAV subfamily. Those genes were also predicted to response to abiotic stress by Go annotation (Table 4). The expression levels of AP2/ERF genes were analyzed under different abiotic stresses.

**Table 4:** Selected AP2/ERF genes putatively related to stress responses by GO annotation.

Gene ID	Subfamily	Annotation
*Pc16182*	ERF	response to cold (GO:0009409); response to water deprivation (GO:0009414); response to abscisic acid stimulus (GO:0009737)
*Pc16943*	ERF	response to water deprivation (GO:0009414); response to abscisic acid stimulus (GO:0009737); response to freezing (GO:0050826)
*Pc24931*	ERF	response to water deprivation (GO:0009414); response to salt stress (GO:0009651); response to freezing (GO:0071497)
*Pc32872*	AP2	response to heat (GO:0009408); response to water deprivation (GO:0009414); response to salt stress (GO:0009651)
*Pc33331*	AP2	response to heat (GO:0009408); response to water deprivation (GO:0009414); response to salt stress (GO:0009651)
*Pc37218*	RAV	no
*Pc41893*	ERF	response to cold (GO:0009409); response to wounding (GO:0009611); response to abscisic acid stimulus (GO:0009737)

**Figure 9:**
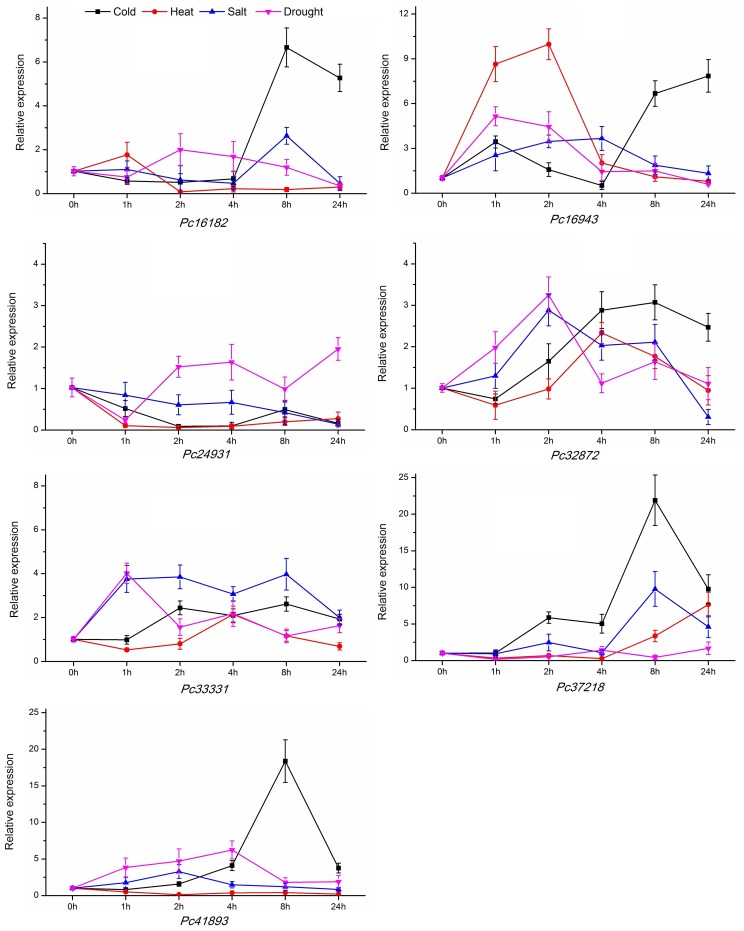
qRT–PCR analysis of AP2/ERF genes in response to different abiotic stresses. Three bio-replicates and tech-replicates were performed. The data are presented as the mean±SD.

As shown in Figure 9, all genes showed sensitivity to cold treatment. The expression level of *Pc24931* rapidly decreased and remained low, whereas those of the other genes increased and peaked after 8 or 24 h. *Pc37218* and *Pc41893* were up-regulated by more than 21 and 18 fold, respectively. *Pc16182*, *Pc24931*, and *Pc41893* were obviously down-regulated under heat stress. *Pc32872*, *Pc33331*, and *Pc37218* were initially down-regulated and then up-regulated. By contrast, *Pc16943* was up-regulated by 9 fold in 2 h and then was rapidly down-regulated. Under salinity stress, *Pc16182*, *Pc16943*, *Pc24931*, and *Pc41893* exhibited minimal or no change in relative expression, but the other four genes increased and showed different levels of sensitivity to salt stress. Under drought treatment, *Pc24931*, *Pc32872*, *Pc33331*, and *Pc41893* were up-regulated, whereas the other genes exhibited no significant change. On the whole, the result was consistent with the annotations.

## Discussion

Current data on the molecular and genetic properties of species in the Apiaceae family are insufficient. Only a few transcriptome databases have been established for celery [29], [30] and carrot [31] in recent years. The lack of reference genomic data has limited parsley research. Transcriptome sequencing is a feasible and economical technology for creating relatively comprehensive sequence data in a short time; this technology has become popular in plant research [35]–[38]. In the present study, 50,161 unigenes were assembled. The obtained sequence data could serve as a basis for further studies on gene cloning, expression analysis, and SSR markers.

More than 60% (31,658 of 50,161) unigenes were annotated by sequence similarity search in seven public databases. Functional annotation can suggest potential gene functions. In our study, qRT-PCR analysis showed that the gene functional annotations were reliable. Others unigenes (approximately 40%) were too short to be annotated. The percentages of the unannotated unigenes were similar to those in rice [62] and tea [63]. Long splicing sequences are among the prerequisites for reliable functional annotation. The insufficient information on the genome and gene functions in the Apiaceae family and the small number of species for referencing have resulted in limited functional annotation.

Molecular marker techniques, such as restriction fragment length polymorphism, random-amplified polymorphic DNA, SNP, and SSR, can be used in genetic diversity analysis. SSR markers are commonly used in genetic linkage map construction and molecular-assisted breeding because of their good repeatability, high reliability, easy operation, and high polymorphism [64], [65]. To our knowledge, this study is the first to report on the SSR markers in parsley. The SSRs motif types, especially the most abundant repeats, were contributed to the evolution of genomes in various organisms [66]. Several research studies have documented GT is the most common type in animal and invertebrates, whereas CT and AT are the most common repeats in plants and insects [67]–[69]. In parsley, the di-nucleotide repeat comprised the largest fraction, while AG/CT and AC/GT are the most abundant motif types. This result agrees with the findings in rice and peach but contradicts with the findings in bread wheat and Medicago truncatula, wherein tri-nucleotide SSRs were found to be the most frequent motif type [70]–[73]. A large number of short repeat sequences are also considered a relatively rapid rate of evolution [74], [75]. Parsley contains a large number of short repeat motifs. We predicted that parsley maybe located on a relatively high level of biological evolution. The formation of mono-, di-, and tri-nucleotide repeat types principally comprised A and T repeat elements, indicating a strong base preference. This preference may be due to the methylation of C residues, which may result in conversion to T [76].

Transcription factors have received more attention from scholars that conducted whole-genomic sequencing and transcriptome sequencing. Drought, high salinity, and extreme temperature are key factors that contribute to crop failure. Previous studies have shown that AP2/ERF transcription factors are related to plant stress response [22], [77]. Two rice ERF genes, *OsERF4a* and *OsERF10a*, confer drought stress tolerance [78]. Some studies showed that the AP2 and RAV subfamilies respond to stress and hormone signals [79], [80]. In the present study, 88 AP2/ERF transcription factors were identified base on parsley transcriptome sequence. We explored the expression levels of AP2/ERF family members belonging to different subfamilies under stress treatments. All selected genes showed different levels of sensitivity to stresses, including *Pc37218*, which was not annotated to response to stress. Some genes from the same subfamily differed in expression. Plant stress tolerance is controlled by multiple genes, and further studies are required to identify the complex regulatory networks of these AP2/ERF genes in parsley.

## Supporting Information

Figure S1 **Phenotypes of** ***Petroselinum crispum*** **and** ***Apium graveolens*** **.**
(TIF)

Table S1 **qRT–PCR primer sequences and the subfamily of selected AP2/ERF genes.**
(XLS)

Table S2 **Gene annotation by seven public databases.**
(XLS)

Table S3 **Gene list in each family of transcription factors.**
(XLS)

Table S4 **Transcript expression level of all unigenes in the curly parsley library.** Gene-ID: gene ID number; Length: gene length; Depth: the average depth of gene; Coverage: coverage of genes; RPKM (reads per kilobase of exon model per million mapped reads): abundance of gene expression; Total reads: the number of reads that hit other genes; Unique reads: the number of reads that hit only one reference gene; Multi reads: the number of reads that hit multiple locations.
(XLS)
