# Supplementary figures and images for: De Novo Transcriptome Sequence Assembly and Identification of AP2/ERF Transcription Factor Related to Abiotic Stress in Parsley (Petroselinum crispum)
Source: PLoS One. 2014 Sep 30;9(9):e108977. doi: 10.1371/journal.pone.0108977 (PMC4182582; doi:10.1371/journal.pone.0108977)

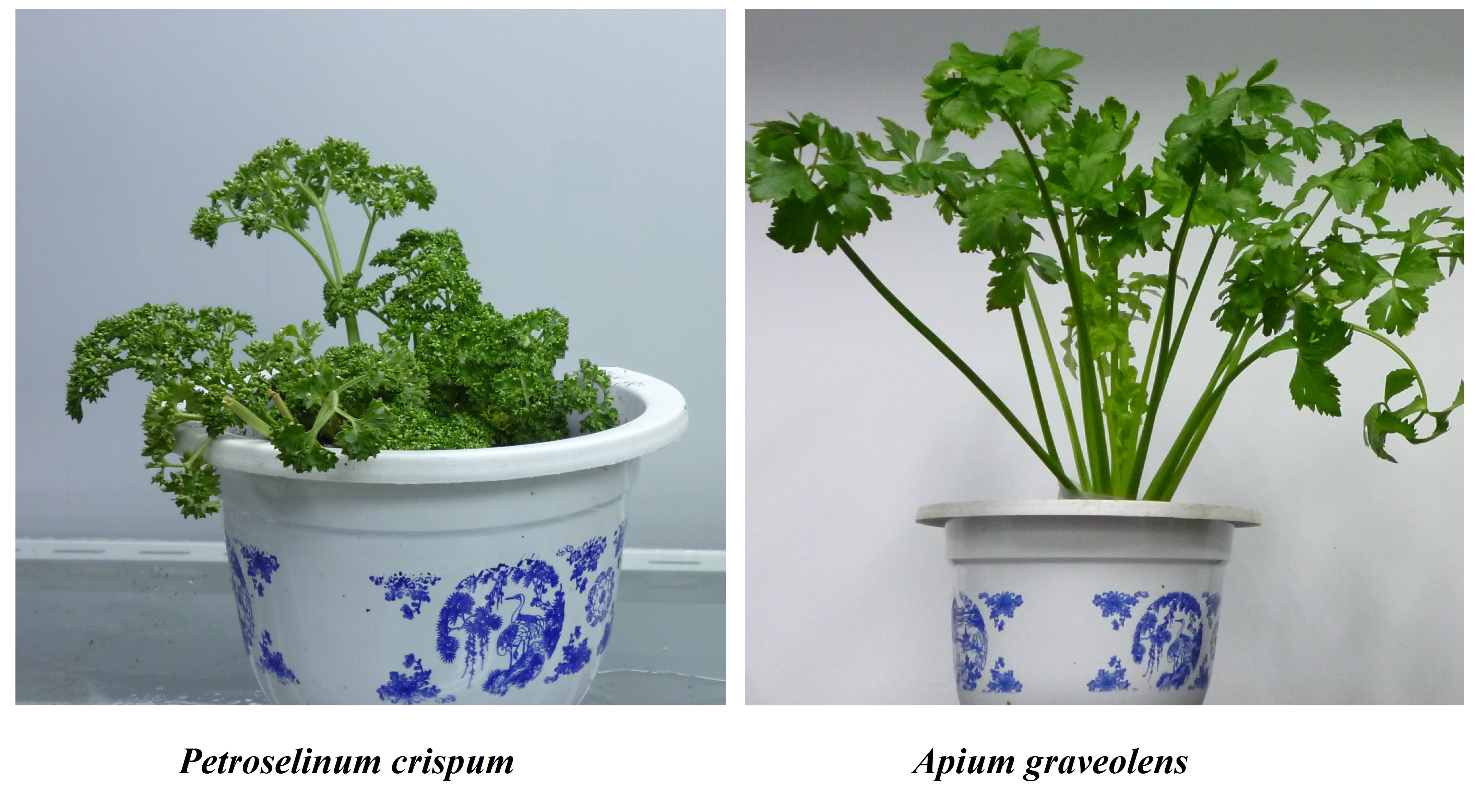

Supplement: Figure S1 — Phenotypes of Petroselinum crispum and Apium graveolens . (TIF) [file pone.0108977.s001.tif]
